# Supplementary figures and images for: Improved accuracy of breast volume calculation from 3D surface imaging data using statistical shape models
Source: PLoS One. 2020 Nov 24;15(11):e0233586. doi: 10.1371/journal.pone.0233586 (PMC7685503; doi:10.1371/journal.pone.0233586)

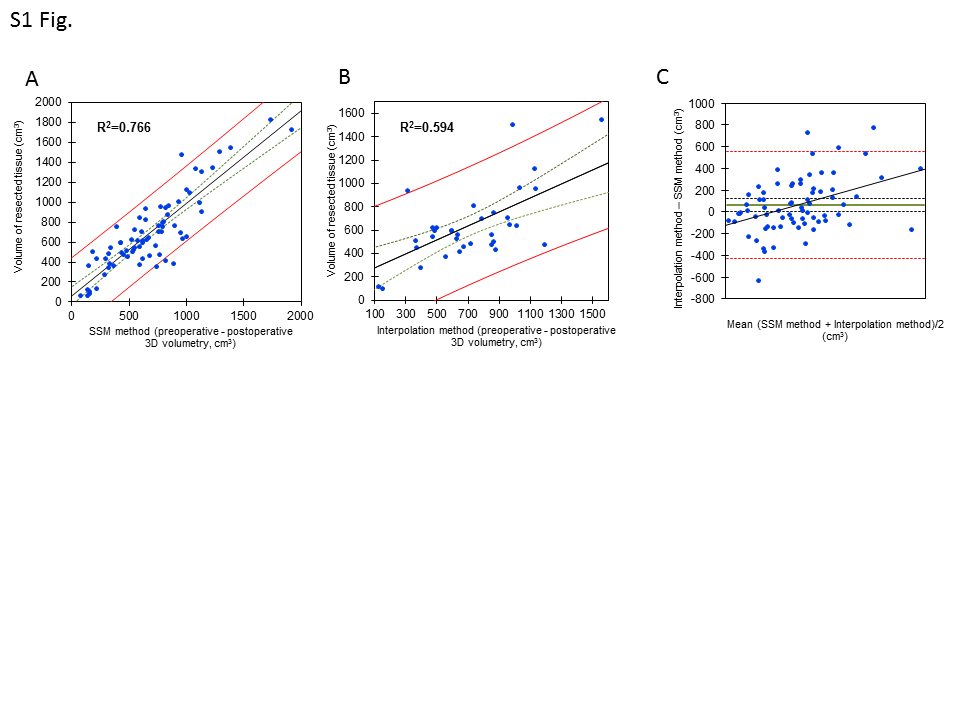

Supplement: S1 Fig — The correlation of the resection weight in breast reduction surgery and the difference between the preoperative and postoperative scans (N = 36 patients, 72 breasts) was found to be higher when the posterior border was interpolated with the SSM-method (A, R2 = 0.766) as compared to interpolation which relied on edges of the breast (B, R2 = 0.594). In this analysis, the latest possible followup timepoint was selected, which means that the followup timepoints are heterogenious. abland-altman plot is drawn comparing the SSM-based and the interpolation method (C). The plot shows some values exceeding the 95% confidence interval. Furthermore, the values seem to cluster at low values and again some kind of logistic regression is noticeable so that we state difference between the comparised methods. When comparing the two methods, we see that with a total p-value of 0.824 in TOST, the equivalence between the methods is rejected, suggesting a significant difference between the SSM-based and the interpolation method. The green lines signify 25% and the red lines 95% confidence intervals. (TIF) [file pone.0233586.s001.tif]

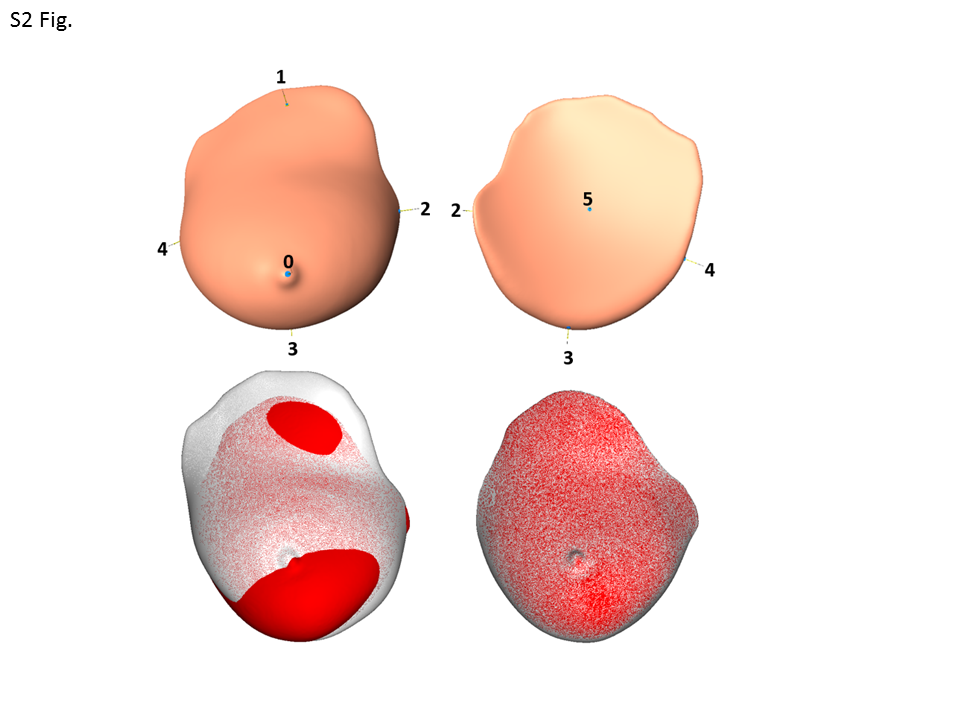

Supplement: S2 Fig — While the first part of the study was focused on creating an SSM of the thoracic wall (Fig 2), we aimed at designing an SSM of the breast for purpose of direct breast volumetry. This model already contains the SSM-based prediction of the posterior breast border and greatly simplifies the workflow, since it becomes sufficient to mark the points on the scan of breast (S3 Fig), instead of having to mark the whole thorax. Samples for building up the model are the estimated breasts of patients participating in this study. Furthermore, all pre- and postoperatively measured patients were included in the model, so that more data than just indirect volumetry were available, thus increasing the statistical coverage of the SSM. Overall, there are 130 samples per page.The above images show the placement of the points for the registration of the samples by a free form deformation based on 6 individually set landmarks for initial rotation. Model building was performed analogous to the SSM of the thoracic wall: Regularization was achieved by a Gaussian Process model that was created from a generic breast template. To allow for shapes outside the model space, a weighted average between the free form deformation and the model was calculated, with the model weights decreasing by each iteration (to 90% of the weight of the previous iteration). The lower two images are illustrating the the elastic registration process. Again we get shapes where each point is in correspondence to each other and we are able to define the covariance matrix which is essential generating a SSM. The procedure has to be done seperately for the left and the right breast. (TIF) [file pone.0233586.s002.tif]

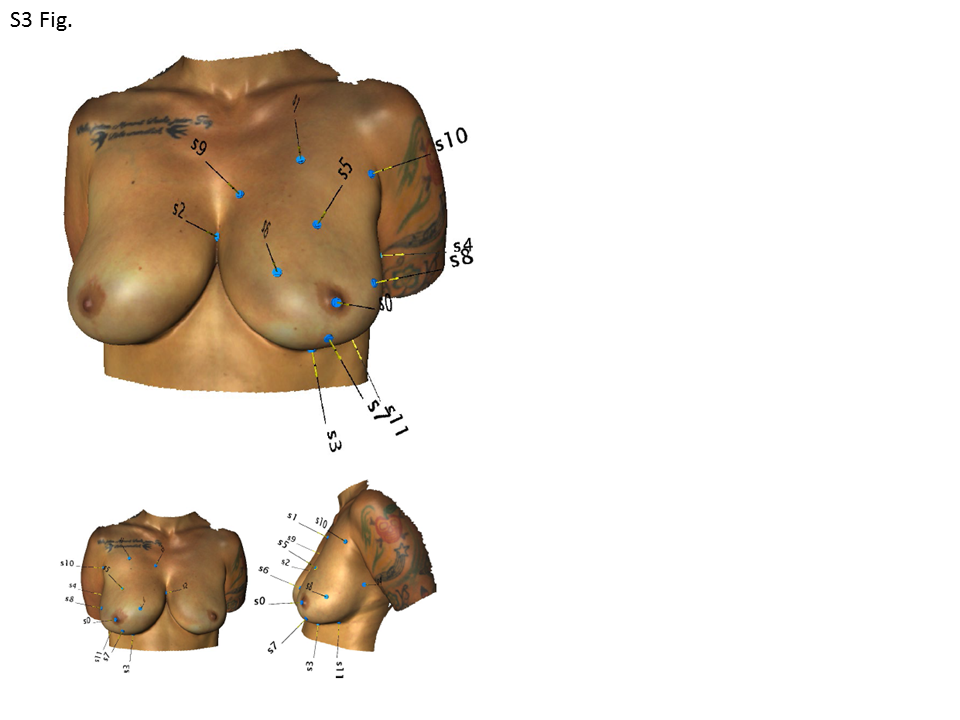

Supplement: S3 Fig — These are the basis for the merge of the 3D scan of the patient with the statistic form model, leading to a 3D breast image with closed posterior border. Manual placement of the points renders this step voulnerable to interuser variability. To reduce the interobserver error and semi-automatic identify the configuration with the closest point distribution, an iterative closest point algorithm (ICP) is run. Afterwards 20 fitting steps are performed by reducing the MSE (mean squared error) from model to target with subsequential elastic registration to achieve a better fit. Anatomical definitions of the points are: 0 –nipple, 1 –cranial breast border (curved extension of the preaxillary fullness), 2 –medial breast border, 3 –caudal border (in the submammary fold), 4 –lateral border of the breast (usually the anterior axillary line), 5 –point halfway between 0 and 1, 6—point halfway between 0 and 2, 7—point halfway between 0 and 3, 8 point halfway between 0 and 4, 9 –mediocranial border (point halfway on the curved line between 1 and 2), 10 –laterocranial border (breast border in the anterior axillary line), 11 –laterocaudal border (point halfway between 3 and 4). (TIF) [file pone.0233586.s003.tif]

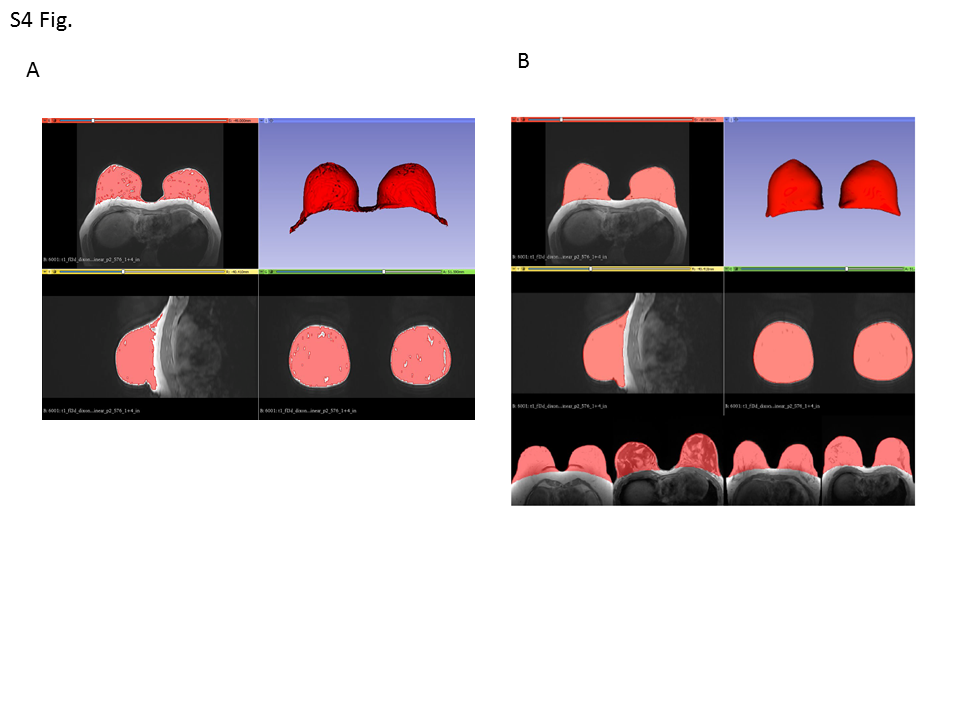

Supplement: S4 Fig — To process the MRI data we used 3D slicer to perform a Otsu-threshold segmentation in the gray scale range from 200 (lower threshold) to 700 or maximum value if below 700 (upper threshold) (A). The resulting segmentation was then smoothed using in 3D slicer implemented Gaussian filters with a 10 mm kernel in the threshold range from 50 to 700. Subsequently and the edges were dilated with a 10 mm kernel in the threshold range from 50 to 700 too. The result is a manually adjusted segmentation with a boundary to the chest posterior wall at skin level (B). (TIF) [file pone.0233586.s004.tif]
