# Supplementary material for: Improved accuracy of breast volume calculation from 3D surface imaging data using statistical shape models
Source: PLoS One. 2020 Nov 24;15(11):e0233586. doi: 10.1371/journal.pone.0233586 (PMC7685503; doi:10.1371/journal.pone.0233586)
Supplement: S1 Table — (DOCX) [file pone.0233586.s005.docx]

**S1 Table**

| Patient_id | Timepoint | PCA method (cm^3^) | Interpolation  method (cm^3^) | BMI (kg/m^2^) | Volume resected (cm^3^) |
| --- | --- | --- | --- | --- | --- |
| **1** | **1y** | **522.81** | **534.58** | **30.47** | **626.17** |
| **1** | **1y** | **420.91** | **401.51** | **30.47** | **598.13** |
| 2 | 2w | 1127.71 | 1105.14 | 35.20 | 1310.28 |
| 2 | 2w | 1226.55 | 1366.05 | 35.20 | 1345.79 |
| 3 | 5w | 139.37 | 55.72 | 22.66 | 60.75 |
| 3 | 5w | 71.61 | -9.79 | 22.66 | 65.42 |
| 4 | 1y | 645.33 | 617.88 | 35.26 | 824.30 |
| 4 | 1y | 547.99 | 207.64 | 35.26 | 727.10 |
| 5 | 5w | 897.35 | 845.21 | 31.22 | 764.15 |
| 5 | 5w | 1112.84 | 1650.46 | 31.22 | 1000.00 |
| **6** | **1y** | **1383.49** | **1696.92** | **47.07** | **1546.73** |
| **6** | **1y** | **1287.69** | **1170.38** | **47.07** | **1508.41** |
| **7** | **1y** | **478.32** | **329.73** | **20.80** | **514.95** |
| **7** | **1y** | **482.45** | **347.44** | **20.80** | **455.14** |
| **8** | **6m** | **842.82** | **1033.22** | **34.53** | **967.92** |
| **8** | **6m** | **999.37** | **964.51** | **34.53** | **652.83** |
| **9** | **6m** | **613.16** | **875.38** | **28.58** | **435.51** |
| **9** | **6m** | **282.88** | **396.17** | **28.58** | **276.64** |
| 10 | 5w | 1029.31 | 949.24 | 34.29 | 1099.07 |
| 10 | 5w | 837.52 | 727.04 | 34.29 | 881.31 |
| 11 | 5w | 1729.35 | 2127.47 | 49.55 | 1828.97 |
| 11 | 5w | 1917.86 | 1760.65 | 49.55 | 1728.97 |
| **12** | **6m** | **136.69** | **126.91** | **20.55** | **121.50** |
| **12** | **6m** | **154.66** | **148.92** | **20.55** | **102.80** |
| **13** | **6m** | **726.19** | **629.76** | **19.03** | **566.04** |
| **13** | **6m** | **791.47** | **866.15** | **19.03** | **754.72** |
| 14 | 1y | 607.22 | 682.58 | 28.63 | 703.74 |
| 14 | 1y | 755.64 | 773.27 | 28.63 | 767.29 |
| **15** | **6m** | **794.83** | **738.42** | **27.66** | **811.21** |
| **15** | **6m** | **998.95** | **1129.41** | **27.66** | **1130.84** |
| 16 | 2w | 218.25 | 232.42 | 29.78 | 432.08 |
| 16 | 2w | 181.35 | 247.57 | 29.78 | 503.77 |
| 17 | 5w | 363.93 | 406.97 | 26.44 | 368.22 |
| 17 | 5w | 317.63 | 278.07 | 26.44 | 342.99 |
| 18 | 1y | 737.01 | 779.02 | 27.61 | 358.88 |
| 18 | 1y | 885.52 | 1250.66 | 27.61 | 385.05 |
| 19 | 2w | 148.46 | 311.64 | 30.82 | 363.55 |
| 19 | 2w | 325.91 | 435.86 | 30.82 | 386.92 |
| 20 | 5w | 155.23 | 139.20 | 20.94 | 83.18 |
| 20 | 5w | 215.69 | 453.80 | 20.94 | 132.71 |
| **21** | **1y** | **573.81** | **210.45** | **33.66** | **612.26** |
| **21** | **1y** | **446.81** | **1179.23** | **33.66** | **477.36** |
| 22 | 5w | 934.02 | 645.70 | 42.16 | 1009.35 |
| 22 | 5w | 820.25 | 1408.41 | 42.16 | 949.53 |
| 23 | 1y | 1131.61 | 1198.20 | 29.72 | 906.54 |
| 23 | 1y | 962.65 | 872.32 | 29.72 | 635.51 |
| 24 | 2w | 589.52 | 1128.75 | 29.24 | 849.06 |
| 24 | 2w | 792.92 | 653.11 | 29.24 | 792.45 |
| **25** | **6m** | **779.35** | **954.78** | **26.81** | **707.55** |
| **25** | **6m** | **663.81** | **1010.63** | **26.81** | **641.51** |
| **26** | **1y** | **590.43** | **537.15** | **19.61** | **377.36** |
| **26** | **1y** | **816.87** | **807.26** | **19.61** | **415.09** |
| **27** | **6m** | **774.93** | **1134.49** | **27.78** | **953.27** |
| **27** | **6m** | **638.60** | **312.99** | **27.78** | **939.25** |
| **28** | **1y** | **765.33** | **985.17** | **28.28** | **480.19** |
| **28** | **1y** | **681.08** | **623.73** | **28.28** | **462.26** |
| **29** | **1y** | **531.72** | **774.98** | **25.34** | **502.83** |
| **29** | **1y** | **611.71** | **699.91** | **25.34** | **618.87** |
| **30** | **1y** | **317.85** | **710.74** | **28.67** | **485.98** |
| **30** | **1y** | **591.34** | **891.13** | **28.67** | **560.75** |
| 31 | 2w | 386.93 | 646.88 | 29.37 | 761.68 |
| 31 | 2w | 293.54 | 469.63 | 29.37 | 437.38 |
| 32 | 2w | 649.99 | 17.49 | 25.10 | 624.30 |
| 32 | 2w | 755.81 | 871.07 | 25.10 | 708.41 |
| **33** | **1y** | **545.26** | **402.20** | **33.12** | **546.23** |
| **33** | **1y** | **537.72** | **800.02** | **33.12** | **527.36** |
| **34** | **6m** | **616.28** | **481.67** | **24.01** | **594.34** |
| **34** | **6m** | **947.74** | **788.18** | **24.01** | **698.11** |
| 35 | 1y | 340.22 | 118.79 | 27.04 | 546.73 |
| 35 | 1y | 436.72 | 177.11 | 27.04 | 498.13 |
| 36 | 2w | 1077.59 | 1855.94 | 37.76 | 1336.79 |
| 36 | 2w | 953.57 | 1157.77 | 37.76 | 1475.47 |

*2w = 2 weeks; 6w = 6 weeks; 6m = 6 months; 1y = 1 year; PCA = principal component analysis; BMI = body-mass-index*
