# Supplementary material for: Improved accuracy of breast volume calculation from 3D surface imaging data using statistical shape models
Source: PLoS One. 2020 Nov 24;15(11):e0233586. doi: 10.1371/journal.pone.0233586 (PMC7685503; doi:10.1371/journal.pone.0233586)
Supplement: S2 Table — (DOCX) [file pone.0233586.s006.docx]

**S2 Table**.

|  | (1) | (2) | (3) |
| --- | --- | --- | --- |
| VARIABLES | Model 1 | Model 2 | Model 3 |
|  |  |  |  |
| PCA method | 1.016*** | 0.825*** | 0.586* |
|  | (0.107) | (0.112) | (0.338) |
| BMI |  | 13.99*** | 7.074 |
|  |  | (4.361) | (10.21) |
| Interaction |  |  | 0.00781 |
|  |  |  | (0.0104) |
| Constant | -27.99 | -295.2*** | -95.13 |
|  | (75.69) | (106.6) | (287.4) |
|  |  |  |  |
| Observations | 34 | 34 | 34 |
| R-squared | 0.737 | 0.802 | 0.806 |

*Standard errors in parentheses; BMI = body-mass-index;*

*PCA = principal component analysis; *** p<0.01*
