# Supplementary material for: Improved accuracy of breast volume calculation from 3D surface imaging data using statistical shape models
Source: PLoS One. 2020 Nov 24;15(11):e0233586. doi: 10.1371/journal.pone.0233586 (PMC7685503; doi:10.1371/journal.pone.0233586)
Supplement: S3 Table — (DOCX) [file pone.0233586.s007.docx]

**S3 Table**.

|  | (1) | (2) | (3) |
| --- | --- | --- | --- |
| VARIABLES | Model 1 | Model 2 | Model 3 |
|  |  |  |  |
| Interpolation method | 0.599*** | 0.378*** | 0.121 |
|  | (0.128) | (0.127) | (0.413) |
| BMI |  | 21.96*** | 14.53 |
|  |  | (6.226) | (12.98) |
| Interaction |  |  | 0.00858 |
|  |  |  | (0.0131) |
| Constant | 215.3** | -244.1 | -33.37 |
|  | (99.76) | (155.9) | (358.3) |
| Observations | 34 | 34 | 34 |
| R-squared | 0.405 | 0.575 | 0.581 |

*Standard errors in parentheses; BMI = body-mass-index;*

*PCA = principal component analysis;* *** p<0.01, ** p<0.05
