# Supplementary material for: Improved accuracy of breast volume calculation from 3D surface imaging data using statistical shape models
Source: PLoS One. 2020 Nov 24;15(11):e0233586. doi: 10.1371/journal.pone.0233586 (PMC7685503; doi:10.1371/journal.pone.0233586)
Supplement: S4 Table — (DOCX) [file pone.0233586.s008.docx]

**S4 Table**

|  | (1) | (2) | (3) |
| --- | --- | --- | --- |
| VARIABLES | Model 1 | Model 2 | Model 3 |
|  |  |  |  |
| PCA method | 0.928*** | 0.676*** | 0.791*** |
|  | (0.0613) | (0.0672) | (0.191) |
| BMI |  | 20.29*** | 23.58*** |
|  |  | (3.542) | (6.197) |
| Interaction |  |  | -0.00352 |
|  |  |  | (0.00544) |
| Constant | 62.68 | -369.5*** | -468.8*** |
|  | (46.90) | (84.85) | (175.5) |
|  |  |  |  |
| Observations | 72 | 72 | 72 |
| R-squared | 0.766 | 0.842 | 0.843 |

*Standard errors in parentheses; BMI = body-mass-index;*

*PCA = principal component analysis; *** p<0.01*
