# Supplementary material for: Improved accuracy of breast volume calculation from 3D surface imaging data using statistical shape models
Source: PLoS One. 2020 Nov 24;15(11):e0233586. doi: 10.1371/journal.pone.0233586 (PMC7685503; doi:10.1371/journal.pone.0233586)
Supplement: S5 Table — (DOCX) [file pone.0233586.s009.docx]

**S5 Table**. Ordinary least squares analysis on 36 patients (second group = latest available follow up) showing the correlation of the resection weight (dependent variable y) with the volume estimation using the interpolation method (x_1_), body-mass-index (x_2_), and an interaction term (x_1_ * x_2_).

|  | (1) | (2) | (3) |
| --- | --- | --- | --- |
| VARIABLES | Model 1 | Model 2 | Model 3 |
|  |  |  |  |
| Interpolation method | 0.641*** | 0.388*** | 0.291 |
|  | (0.0633) | (0.0645) | (0.201) |
| BMI |  | 27.50*** | 24.48*** |
|  |  | (4.333) | (7.359) |
| Interaction |  |  | 0.00300 |
|  |  |  | (0.00591) |
| Constant | 215.2*** | -413.8*** | -324.5 |
|  | (55.14) | (108.5) | (206.9) |
|  |  |  |  |
| Observations | 72 | 72 | 72 |
| R-squared | 0.594 | 0.744 | 0.745 |

*Standard errors in parentheses; BMI = body-mass-index;*

*PCA = principal component analysis; *** p<0.01*
